# Error Analysis of Magnetohydrodynamic Angular Rate Sensor Combing with Coriolis Effect at Low Frequency

**DOI:** 10.3390/s18061921

**Published:** 2018-06-13

**Authors:** Yue Ji, Mengjie Xu, Xingfei Li, Tengfei Wu, Weixiao Tuo, Jun Wu, Jiuzhi Dong

**Affiliations:** 1 Key Laboratory of Advanced Electrical Engineering and Energy Technology, Tianjin Polytechnic University, Tianjin 300387, China; 2 School of Instrument Science and Opto-Electronics Engineering, Hefei University of Technology, Hefei 230009, China; xumj@hfut.edu.cn; 3 State Key Laboratory of Precision Measuring Technology and Instruments, Tianjin University, Tianjin 300072, China; lixf@tju.edu.cn (X.L.); wtf@tju.edu.cn (T.W.); tuoweixiao@tju.edu.cn (W.T.); 4 Aviation Engineering Institute, Civil Aviation University of China, Tianjin 300300, China; j_wu@cauc.edu.cn; 5 Advanced Mechatronics Equipment Technology Tianjin Area Major Laboratory, Tianjin Polytechnic University, Tianjin 300160, China; dongjiuzhi@hotmail.com

**Keywords:** magnetohydrodynamic, Coriolis effect, low frequency expansion, angular rate sensor

## Abstract

The magnetohydrodynamic (MHD) angular rate sensor (ARS) with low noise level in ultra-wide bandwidth is developed in lasing and imaging applications, especially the line-of-sight (LOS) system. A modified MHD ARS combined with the Coriolis effect was studied in this paper to expand the sensor’s bandwidth at low frequency (<1 Hz), which is essential for precision LOS pointing and wide-bandwidth LOS jitter suppression. The model and the simulation method were constructed and a comprehensive solving method based on the magnetic and electric interaction methods was proposed. The numerical results on the Coriolis effect and the frequency response of the modified MHD ARS were detailed. In addition, according to the experimental results of the designed sensor consistent with the simulation results, an error analysis of model errors was discussed. Our study provides an error analysis method of MHD ARS combined with the Coriolis effect and offers a framework for future studies to minimize the error.

## 1. Introduction

Lasing and imaging systems are increasingly required by new applications in the commercial, defense, and communication worlds. With these new applications, in-obit vibration throughout a wide bandwidth (DC-1K Hz) can be induced by external disturbance and various motions of the loads on the mobile carrier [1]. The unwanted line-of-sight (LOS) jitter can seriously affect the performance of the systems [2]. In order to achieve the mission objectives, their LOS system must provide precise pointing and tracking capabilities with suppression of jitter to levels of 1–3 micro-radians (rms) or less [3], especially in deep space laser communications [4] and directed energy weapons [5]. As a result, the requirements for the extremely accurate measurement of the LOS jitter are often specified in micro-radians or even nano-radians in wide bandwidth (DC-1K Hz) [6]. The magnetohydrodynamic (MHD) angular rate sensor (ARS) is developed for its unique advantage of extremely low noise level in ultra-wide bandwidth [7] and demonstrated in many systems, such as the measurement of angular acceleration in an impact environment [8], the Relay Mirror Experiment [9], the Advanced Land Observing Satellite [10], and the Mars Laser Communication Demonstration program [11]. 

However, the MHD ARS does not possess the capability of measurement at low frequency (<1 Hz), especially the constant inertial angular rate [12], which is essential for precision LOS pointing and wide-bandwidth LOS jitter suppression [13,14]. Martin et al. [15] investigated whether the differential of MHD ARS could be used to measure head angular acceleration in impact testing. The results showed that the differential of angular velocity deduced by MHD ARS are consistent with the vibratory response of the pendulum for various magnitudes of angular acceleration, but inconsistent due to its poor performance at low frequency. Merkle et al. [16] showed that the noise level of MHD ARS was better than two other rate gyroscopes while the MHD ARS’s discrepancy in the experiment from the benchmark was larger due to its drift. Farr et al. [17] discussed the application of MHD ARS in the rejection of angular disturbances in the flight laser transceiver and stated that the sensor’s poor low-frequency noise performance could depress the combination measurement results. Thus, the limitation of MHD ARS at low frequency implies a large drift rate and has a negative impact on the sensor’s tracking accuracy and application range. 

Substantial explorations have been carried out to expand the MHD ARS bandwidth at low frequency, especially focusing on combining it with the conventional gyroscope. Laughing et al. [18] proposed the design of a blending filter to combine the high-frequency MHD ARS sensor measurement with the low frequency rate estimate by a conventional gyroscope. The method was based on the extremely accurate estimation of two sensors’ spectral characteristics and the disturbances that cause LOS jitter [19]. Pinney et al. [20] provided a detailed analysis of the predicted angular position error as a function of the measurement duration to compensate the sensor’s drift. Iwata et al. [21] described an implementation of a Wiener-filter-based complementary filtering and a Kalman filtering, using data from a star tracker, gyroscopes, and MHD ARS. However, the procedure was hard to guarantee in real-time and adapt to complex application environments. Burnside et al. [22] designed a blended tracking concept by optical tracking sensors combing with MHD ARS to provide a composite pointing reference. The frequency and noise characteristics of the blended zone were not ideal in the dynamic experiment. Reviewing these existing combination measuring methods, we can find that the low-frequency compensation methods make strict demands on the real time and accuracy of the combinatorial technique and increase the complexity of the system. It is essential to alter the sensor’s physical construction to sense a low-frequency rate without affecting its characteristic at high frequency.

In contrast to the mentioned approaches, Laughing et al. [23] presented a design concept of a modified MHD ARS combining the Coriolis effect with the MHD effect to extend the measurement scope throughout the whole bandwidth. However, no further error analysis and experimental data have been given until now. In our previous paper [24], we designed a device to study the radial velocity generation for introducing the Coriolis effect in the MHD ARS, which is critical for the combination of two effect. In this paper, the error analysis of the modified MHD ARS is studied by numerical simulation and experiment. This paper is organized as follows:

In Section 2, the modeling and simulation method is established. In Section 3, the design of the sensor, as well as the numerical simulation results are presented and analyzed in detail. The experimental results of the sensor are illustrated and discussed in Section 4. Finally, the paper ends with a conclusion in Section 5.

## 2. Modeling and Simulation Method

In this part, the basic working principle of the modified MHD ARS is illustrated based on a simplified model. In order to explain the Coriolis effect generation method, the design of the fluid channel is presented. Basic governing equations and a solution method in the numerical simulation are given. In addition, a comprehensive solving method based on the magnetic and electric interaction methods is proposed to study the physical process of the MHD pump and MHD sensing in the modified MHD ARS.

### 2.1. Modeling of the Modified MHD ARS Combing with Coriolis Effect (Abbreviated as C-MHD ARS)

#### 2.1.1. The Working Principle of C-MHD ARS 

**Figure 1:**
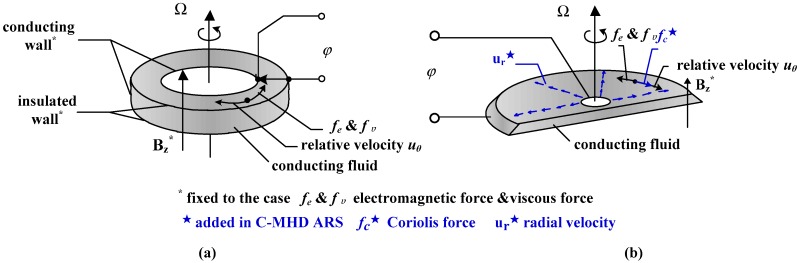
Description of the annular channel in the basic magnetohydrodynamic angular rate sensor MHD ARS and C-MHD ARS. (**a**) The basic one (**b**) the C-MHD ARS (with Coriolis Effect).

The MHD ARS, which measures angular velocity, can also provide angular position and acceleration by integrating or differentiating the output with respect to time. Then, the linear accelerometers can be derived, which is important for navigation systems [25,26]. The basic MHD ARS is mainly based on the magnetohydrodynamic effect as shown in Figure 1a. The magnetic field B_z_* is fixed to a cylinder case and the whole attached to a body whose inertial angular motions are measured. As the case rotating with angular velocity Ω, the inertial-fixed fluid moves through the case-fixed magnetic field B_z_* with relative velocity *u_θ_*. The top and bottom of the fluid annulus are insulated, while the inner and outer perimeters of the fluid annulus in contact with electrodes to measure the voltage φ. The electromagnetic force *f_e_* and viscous force *f_υ_* are always opposite to the direction of the relative velocity. Assuming there is no secondary flow and induced magnetic field, the basic MHD ARS transfer function can be approximately simplified as [12,27]:

(1) $$\left|\frac{\varphi (s)}{\Omega (s)}\right|=\frac{B_{z}WRs}{s+\sigma {B_{0}}^{2}/\rho +\nu /h^{2}}=\frac{B_{z}WRs}{s+\left(\nu /h^{2}\right)(1+Ha^{2})}$$

where, the conducting fluid is assumed to be viscous with the kinematic viscosity coefficient *ν*, incompressible, and homogeneous with a constant electrical conductivity σ and mass density ρ. The top and bottom sides are parallel insulating plates with height *h*. Inside and outside walls are concentric conducting cylinders with radii *r_i_* and *r_o_*. Here, the equivalent radius *R* = (*r_i_* + *r_o_*)/2 is defined and *W* = *r_o_* − *r_i_* is the width of the annular channel, and Ha is the Hartmann number ($\mathrm{Ha}=\sqrt{\sigma {B_{0}}^{2}h^{2}/\nu \cdot \rho }$), which gives the ratio electromagnetic force *f_e_* to viscous force *f_υ_*.

When rotating with high-frequency vibrations, the two forces *f_e_* and *f_υ_* are small enough to be ignored and the voltage φ across the inertial-fixed fluid is linearly related to the inertial angular velocity Ω. While the angular velocity frequency is near or below the cutoff frequency, the electromagnetic induction is weakened.

Compared with the basic one in Figure 1a, the C-MHD ARS introduces a radial velocity *u_r_* to accelerate the relative circumferential velocity *u_θ_* due to the Coriolis effect at low frequency, as shown in Figure 1b. The Coriolis force *f_c_* is induced by the radial flow as the fluid rotating with the walls of the sensor case, whereas the MHD effect dominates at high frequency for the inertial-fixed fluid. The measurement through the whole bandwidth is achieved by combing the Coriolis effect at low frequency with MHD effect at high frequency.

The simplified formula can be approximately written as (2) under three assumption. (1) The radial velocity in the annual channel is constant and uniform; (2) The axis and radial velocity can be negligible compared with the circumferential velocity; (3) Only taking the current induced by relative circumferential velocity into consideration and ignoring the induced magnetic fields under the assumption of a small Reynolds number. The specific deduction can be seen in our earlier paper [24].

(2) $$\left|\frac{\varphi \left(s\right)}{\Omega \left(s\right)}\right|=\frac{B_{z}W(R\cdot s+2u_{r})}{s+\sigma B^{2}/\rho +\nu /h^{2}}=\frac{B_{z}WR\left(s+2u_{r}/R\right)}{s+\left(\nu /h^{2}\right)\left(1+Ha^{2}\right)}$$

From the expression, we can draw the conclusion that the expression $\left|\varphi \left(s\right)/\Omega \left(s\right)\right|=B_{z}WR$ can be achieved in the whole pass band when the expression $u_{r}=R/2\cdot \left(\nu /h^{2}\right)\left(1+Ha^{2}\right)$ can be satisfied. Otherwise, the transition of amplitude and phase frequency response at the frequency $f_{z}=\left(1/2\pi \right)\cdot \nu /h^{2}(1+Ha^{2})$ would exist, and then the ratio of the voltage φ and the angular rate Ω would be quite different between low frequency and high frequency. According to the exploration in studying the simple MHD ARS previously, the simplified model cannot reflect the compressive nonlinear and coupled relationship in MHD ARS, especially at low frequency. Thus, further error analysis based on numerical simulation and experiment in this paper will be illustrated.

#### 2.1.2. The Design of Fluid Channel

**Figure 2:**
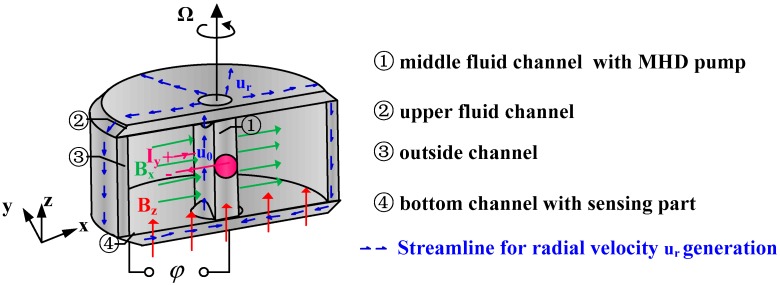
Description of the fluid channel in C-MHD ARS.

The radial rate generation method by a MHD pump has been verified in paper [24] according to the numerical calculation and experimental results of a designed device. Figure 2 presents the streamline for inducing the radial velocity in the annulus and the whole fluid channel of the C-MHD ARS. The fluid in the middle channel is driven along the *z*-axis by the orthogonal magnetic field *B_x_* and electrical current *I_y_*. Since the smaller flux of the middle channel is compared with the outside channel, the gravity is expected to be utilized in the reflux process. The direction of the velocity *u_0_* generated by the MHD pump is advised upward instead of downward. Then the fluid is forced radially outward in the upper channel and travels through the outside channel to the bottom channel. In order to leave room for the energized electric current leads, the upper fluid channel may be incomplete. Therefore, the bottom channel is chosen as the sensing part and the magnetic field *B_z_* is applied to generate the electromotive voltage φ as the case rotating with angular velocity Ω. Thus, the sensing part works as shown in Figure 1b. As it rotates, the Coriolis effect is induced by the radial flow to accelerate the relative circumferential velocity *u_θ_* especially at low frequency.

#### 2.1.3. Basic Governing Equations of C-MHD ARS

The governing equations can be expressed as Equations (3)–(6). With the knowledge of magnetohydrodynamics, the MHD coupling is achieved by additional source terms to the fluid momentum equation. The Navier–Stokes equation for an electrically conducting and incompressible Newtonian fluid can be written as Equation (3). The additional source term in the Navier–Stoke equation is the Lorentz force given as F=J×B. In addition, the conductive fluids such as mercury and Galinstan are almost heavy fluid, so the gravity ρ⋅g should be taken into account. According to the law of conservation of mass, the velocity of homogeneous Newton fluid can be given as Equation (4). Besides, the governing equations include the magnetic diffusion Equation (5) and the expression of magnetic induction intensity in passive field (6) [28,29,30].

(3) $$\rho \frac{du}{dt}=-\nabla p+\left(\nu \cdot \rho \right)\cdot \nabla^{2}u+\frac{1}{\mu }\left(\nabla \times B\right)\times B+\rho \cdot g+J\times B$$

(4) ∇⋅u=0

(5) $$\frac{\partial B}{\partial t}=\frac{1}{\mu \sigma }\nabla^{2}B+\nabla \times \left(u\times B\right)$$

(6) ∇⋅B=0

Magneto-conductivity, total magnetic field, the electric current density, the pressure field, and the velocity field are respectively denoted by *μ*, **B**, **J**, **p** and **u**.

### 2.2. Model Solution Method

#### 2.2.1. Simulation Program and Solution Methods 

To solve this model, commercial software 3D-Magnetostatic and FLUENT coupled with MHD module packaged in ANSYS are adopted. 3D-Magnetostatic is used to calculate the magnetic field distribution of the fluid channel including the one *B_y_* in the MHD pump and the one *B_z_* through the bottom channel. Then the magnetic simulation results are exported to the computation fluid domain in FLUENT as the external magnetic field data. The computation mesh created with ANSYS ICEM is fully structured. The problem involves the moving parts and it is the flow through the moving parts that is of interest. Therefore, we chose the rotating case as the moving reference frame and the relative velocity formulations are applied. The user-defined function (UDF) is used to simulate the time-varying vibration of the case. At each time step, the spatial discretization based on the Green—Gauss approach is performed. Fluxes in momentum and electrodynamics are evaluated by means of second-order upwind discretization. Time integration is performed utilizing first-order implicit scheme.

#### 2.2.2. The Solving Method of Interaction between Flow Field and Electromagnetic Field and Boundary Conditions

In studying the interaction between the flow field and electromagnetic field, it is critical to know the current density **J** due to induction. Since the physical processes of the MHD pump and sensing part are quite different, the different interaction methods and boundary types must be chosen [31,32]. Generally, two approaches may be used to evaluate the current density in FLUENT. One is through the solution of a magnetic induction equation; the other is through solving an electric potential. Boundary conditions in the two methods are set quite different in the MHD module coupled with FLUENT.

In the first approach, the magnetic induction equation is derived from Ohm’s law and Maxwell’s equation. The Equation (5) can be written as:

(7) $$\frac{\partial B}{\partial t}+\left(u\cdot \nabla \right)B=\frac{1}{\mu \sigma }\nabla^{2}B+\left(B\cdot \nabla \right)u,$$

with $B=B_{0}+b$, the induction Equation (7) can be defined as (8) in the conductive medium:

(8) $$\frac{\partial b}{\partial t}+\left(u\cdot \nabla \right)b=\frac{1}{\mu \sigma }\nabla^{2}b+\left(\left(B_{0}+b\right)\cdot \nabla \right)-\left(u\cdot \nabla \right)B_{0},$$

and the current density is given by:

(9) $$j=\frac{1}{\mu }\nabla \times \left(B_{0}+b\right)$$

The boundary conditions of magnetic field at conducting and insulating walls in the first approach are respectively denoted as (10) and (11):

(10) $$\frac{\partial b}{\partial n}=0$$

(11) $$\frac{\partial b}{\partial \tau }=0$$

The first magnetic approach is suitable for the electric induction by the conductive fluid motion under the magnetic field following Faraday’s law. The generation of induced electromotive voltage in the annual bottom sensing channel should be simulated using the first approach. Thus, the boundary of conductive internal and external walls in the bottom sensing part should be given as (10) and the upper and bottom insulating ones can be regarded as (11).

On the other hand, the second approach for the current density is to solve the electric potential equation and calculate the current density using Ohm’s law. For a static field and assuming **b**≪**B_0_**, Ohm’s law can be written as (12), when the magnetic Reynolds number Rem=μσU0l is far less than 1.

(12) j=σ(−∇φ+(u×B))

For sufficiently conducting media, the principle of conservation of the electric charge gives:

(13) ∇⋅j=0

The electric potential equation is therefore given by:

(14) $$\nabla^{2}\varphi =\nabla \cdot \left(u\times B_{0}\right)$$

The boundary conditions of magnetic field at conducting and insulating walls in the second approach are respectively denoted as (15) and (16):

(15) $$\varphi =\varphi_{0}\;\;or\;\;J=J_{0}$$

(16) $$\frac{\partial \varphi }{\partial n}={\left(u\times B\right)}_{boudary}\cdot n$$

Compared with the first magnetic method, the second electric method is applicable for analyzing the conductor’s force and motion driven by of its energized current and magnetic field. The fluid flow driven by the MHD pump in the middle channel Figure 2 should be simulated using the second approach. Thus, the boundary of energized electrodes in the middle channel for the MHD pump should be denoted as (15) and the other insulating ones can be set as (16).

In summary, the simulation of MHD pump for radial velocity generation in FLUENT with the help of MHD module is made using the electric approach according to the Equations (3), (4), (6), (13) and (14) with the boundary conditions (15) and (16). On the other hand, the MHD sensing part is simulated using the magnetic approach according to the Equation (3), (4), (6), (8), and (9) with the boundary conditions (10) and (11).

**Figure 3:**
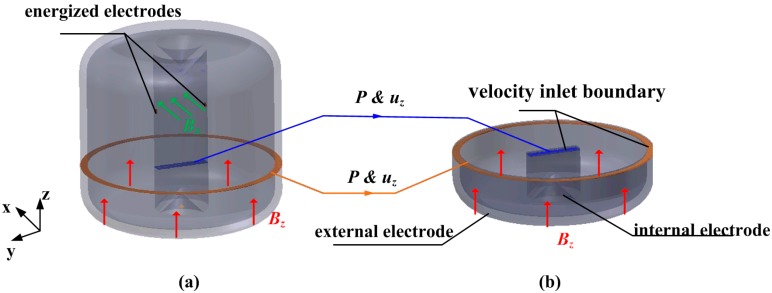
The computation domains with the two interaction solving methods. (**a**) fluid domain using the second electric approach (**b**) fluid domain using the first magnetic approach.

Specifically, the boundary type of energized electrodes in the MHD pump is a fixed electric current condition as **J** = **J_0_** as Equation (15), while the internal and external electrode in the sensing channel should be given as Equation (10) for the unknown induction electromotive force and current. However, only one kind of interaction method can be chosen in the same simulation using the MHD module of FLUENT. To solve the boundary type alternating process, a combined computation method is put forward and illustrated in Figure 3. In the software, the radial velocity driven by the MHD pump is simulated in the whole fluid domain using the second electric approach as shown in Figure 3a. Obviously, the radial velocity distribution in the bottom channel is difficult to export and reuse for the next sensing simulation directly. The stable calculation results of static pressure *P* and velocity *u_z_* on the cross sections are adopted as the velocity inlet boundary to repeat the induced radial velocity in the bottom channel. The cross-plane should be selected in the pumped stability fluid region. Then the first magnetic approach is chosen in the sensing simulation of the fluid domain shown in Figure 3b, in which the radial velocity distribution is reproduced by the velocity inlet in the middle and outside channels. The other walls except internal and external electrodes are set as insulating walls with the boundary condition expressed as (11). At last, the generated voltage of the C-MHD ARS with the designated current in MHD pump can be given.

## 3. Simulation Results and Analysis 

Before the numerical simulation is carried out, the design of the C-MHD ARS has been modified and improved according to the previous simulation and experimented on using fluid and magnetic fields, which is described in this section. As a critical process during the working of the C-MHD ARS, the Coriolis effect induced by the MHD pump is simulated and analyzed in detail. Finally, the simulation results of the C-MHD ARS frequency response are given and the error analysis is discussed.

### 3.1. Description of the Designed C-MHD ARS

**Figure 4:**
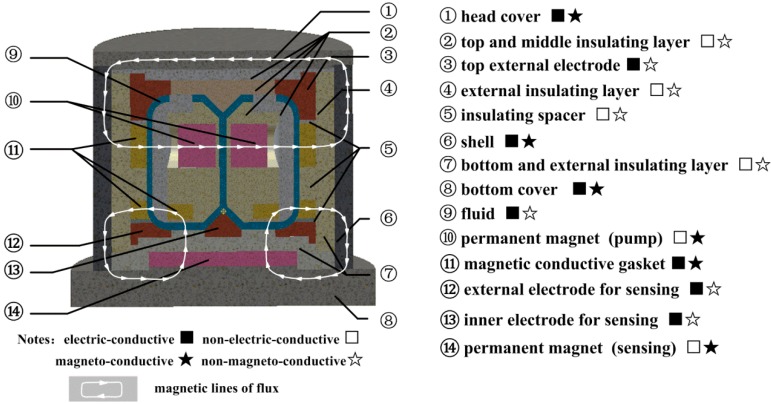
Schematic diagram of the designed C-MHD ARS in simulation and experiment.

**Figure 5:**
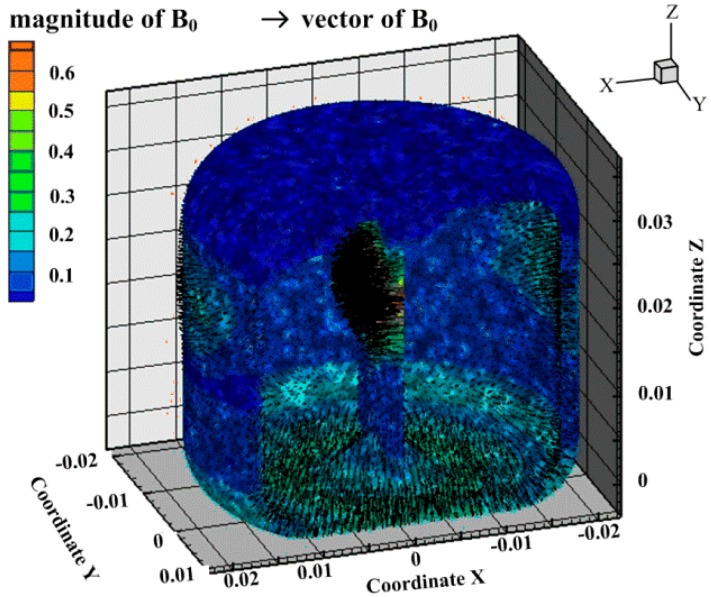
Contour and vector graph of the imposed magnetic field in the fluid channel.

In the previous simulation and experiment on the radial velocity generation by MHD pump in middle channel, it has been confirmed that the thickness and the height of the central channel should be compromised for the velocity rate and steady. Besides, the inhomogeneity of the magnetic field in the sensing channel can bring out the measurement error at the peaks and valleys of an angular vibration. Based on these design experiences concerning the fluid and electromagnetic field, the C-MHD ARS in this paper is designed as shown in Figure 4. The head cover (1), the shell (6), and the bottom shell (8) with high permeability material form a closed magnetic circuit for magnetic shielding. A pair of permanent magnets (10) imposes the magnetic field for the MHD pump in the middle channel. The magnetic field in the bottom sensing channel along the vertical direction is generated by the permanent magnets (14). In the entire structural design, the independence of the magnetic field for MHD pump and the sensing part is critical. Therefore, the magnetic conductive gaskets (11) and the insulating spacers (5) are designed to make the enclosed magnetics lines along the shortest paths plotted. Furthermore, the position of the magnets (10) is above the center of the middle fluid channel to make the magnetic lines of the MHD pump far from the ones for sensing. The imposed magnetic field distribution in the fluid channel is plotted in Figure 5. The average of the magnetic field *B_z_* along axis *z* in the sensing channel is about 0.12 T and the inhomogeneity of the magnetic field is about 52.4%. The parts (12 and 13) acting as the electrodes of the sensing channels are electric-conductive, but non-magneto-conductive, and ensure that the direction of the magnetic field in the fluid is along the vertical axis. Meanwhile, the insulating parts (2 and 7) form the walls of the fluid channel 9 for electric isolation.

As plotted in Figure 4, the fluid wall is composed of different materials at the top, external and bottom channels, which brings out a great challenge for the seal work. Therefore, Galinstan is chosen as the conductive fluid in the C-MHD ARS instead of Hg, for its less volatilization and nontoxicity, to explore the appropriate sealing technology. The physical parameters of Galinstan are listed in Table 1. Clearly, the material physical characteristics can satisfy the sensor environment temperature in the application, but it may be inferior to Hg in lower density and higher viscosity. Besides, the main mechanical structure parameters of the fluid channel are adjusted repeatedly in the simulation according to the experience in the previous paper [33,34] and then given in Table 1.

### 3.2. Simulation Results and Analysis on the MHD Pump

**Figure 6:**
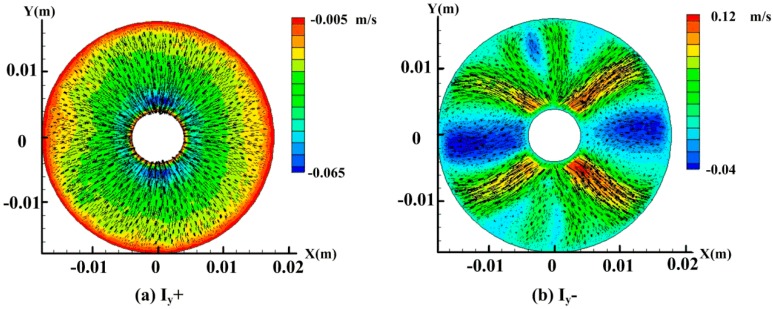
Contours of the radial velocity and the vector graph of velocity on the middle plane *z* = −0.001 m with the imposed current *I_y_* = 3 A and *I_y_* = −3 A in a different direction.

In the previous simulation on the radial velocity generation, it has been confirmed that the fluid flow can be induced above a starting current. In theory, the energized current *I_y_* along the forward or reverse direction can drive the flow to form the circumfluence. However, the homogeneity and stability of the radial velocity *u_r_* in the sensing channel of C-MHD ARS need to be given more attention. To validate, the simulation with the energized current *I* = 3A along different directions is made using the electric approach in the fluid domain, as seen in Figure 3a, without any angular velocity. In the simulation, the mesh coordinate of the sensing bottom channel is along the axis *z*, from −0.002 m to 0 m. We chose the middle plane, *z* = −0.001m, to provide an analysis using the graph of the velocity shown in Figure 6. When the current *I_y_*+ is energized under the orthogonal imposed magnetic field *B_x_*+, as shown in Figure 5, the flow is driven upward in the middle channel and the radial flow in the sensing bottom channel is centripetal. In Figure 6a, the radial velocity is increased along the direction of the centripetal and the velocity of the vector is evenly distributed toward the center. Notably, the relative inward circumferential velocity needs to be more accelerated according to the sensing effect based on the Coriolis effect. On the other hand, when the energized current *I_y_*− is set, the direction of the radial velocity contains inconsistencies. In detail, the vector along the centripetal direction appears near the coordinate *y* = 0, which is quite different from the other positions along the centrifugal direction, as shown in Figure 6b. That is to say, the Coriolis force is confused in this case, which is not suitable to compensate the MHD effect for MHD ARS at low frequency. This may be attributed to the property of the heavy fluid, which makes it difficult for the fluid in the outside channel to overcome the gravity and flow upward evenly. Moreover, the distribution of the velocity vector may be related to the cuboid shape of the MHD pump, which needs to be improved further.

**Figure 7:**
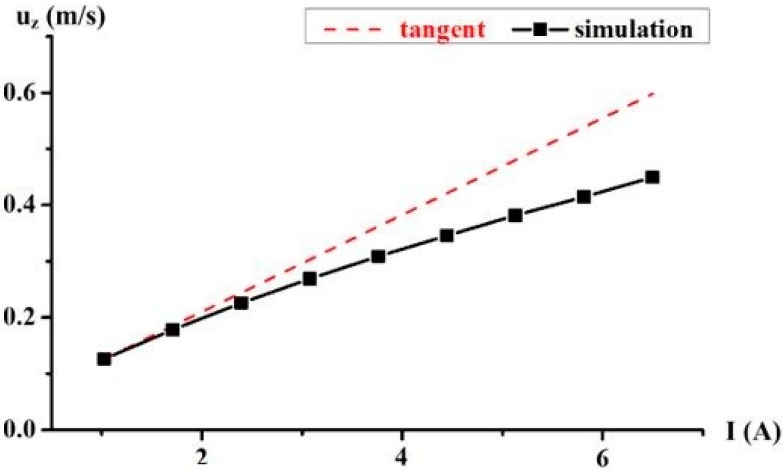
The velocity *u_z_* in the center of the MHD pump with different imposed current *I*.

Likewise, the quantitative analysis of the velocity *u_z_* in the center of the MHD pump is made under a different imposed current *I*. The results are plotted in Figure 7. By the least squares fit, this relationship can be expressed as (17). The tangent is given by the rising speed at the starting current at about *I* = 1 A. With the increase of current, the incremental of the velocity *u_z_* decreases. Naturally, the viscous force is proportional to the velocity and has an obstruction effect on the flow.

(17) $$u_{z}=-0.79644e^{\left(-I/9.1134\right)}+0.83667$$

### 3.3. Simulation Results and Analysis on Coriolis Effect in C-MHD ARS

**Figure 8:**
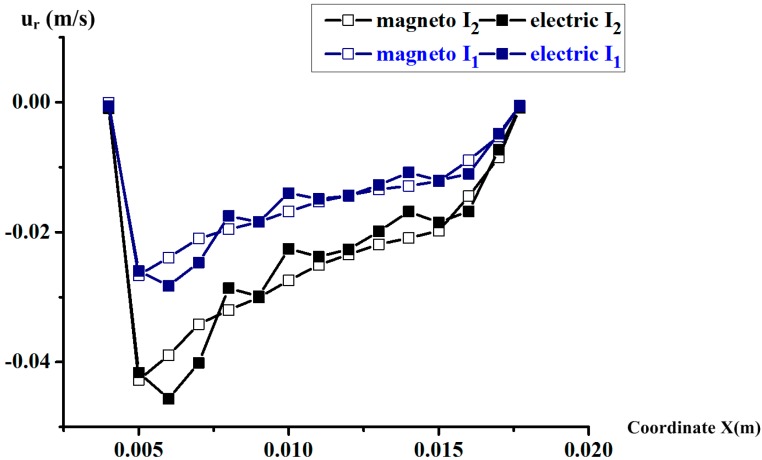
The radial velocity along the axis *x,* respectively, on the plane *z* = −0.001 m using the electric and magnetic method under energized current *I*_1_ = 1.5 A and *I*_2_ = 3 A.

Before we begin the sensing part of the simulation using the magnetic approach in the fluid domain as shown in Figure 3b, it is critical to test the transmission of the radial velocity distribution in the bottom sensing channel. The comparison of the radial velocity along the *x*-axis between the two solving methods is made. The results are plotted in Figure 8, respectively, under *I*_1_ = 1.5 A and *I*_2_ = 3 A. Obviously, the uniformity strongly confirms the previous assumption on the interactive method between the two approaches, as illustrated in Section 2.2. Thus, the next sensing effect analysis using the magnetic approach can be given based on the radial velocity under the current setting in the MHD pump, obtained by the electric method.

**Figure 9:**
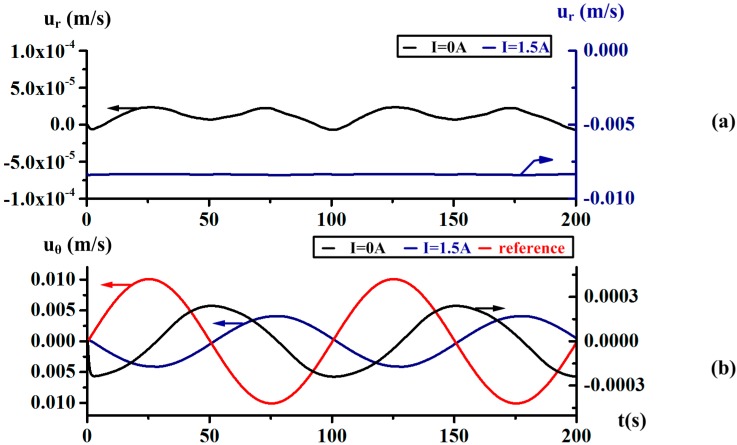
The radial velocity *u_r_* and relative circumferential velocity *u_θ_* of test point *P*(0.010, 0, −0.001) under energized current *I* = 0 A and *I* = 1.5 A when rotating with Ω = sin(2π·0.01·t).

It is known that the Coriolis effect could be induced as the driven radial flow when the case is rotating. The simulation with angular velocity Ω = sin(2π·0.01·t), under energized current *I* = 0 A, and *I* = 1.5 A are made to provide an analysis of the Coriolis effect. In Figure 9, we compare the radial velocity *u_r_* and the relative circumferential velocity *u_θ_* of the test point *P*(0.010, 0, −0.001) in the sensing bottom channel. The velocity curve of the boundary point *P*(0.010, 0, −0.002) is plotted as a reference for comparison. When without energized current, the radial velocity with the order of a few 10^−4^ m/s is negligible. As the energized current is set at 1.5 A, the radial flow along the centripetal direction is induced and its magnitude is about 0.008 m/s. It is apparent that the radial velocity is mainly determined by the MHD pump and is unaffected by angular motion. At the same time, the amplitude of relative circumferential velocity *u_θ_* is increased from 0.0002 m/s to 0.004 m/s. The phase difference between the relative circumferential velocity and the reference signal is about 170° under energized current *I* = 1.5 A, while nearly 90° without energized current. It seems that the relative circumferential motion is accelerated by the energized radial velocity. Thus, the poor performance of the MHD effect at low frequency may be compensated by the Coriolis effect.

**Figure 10:**
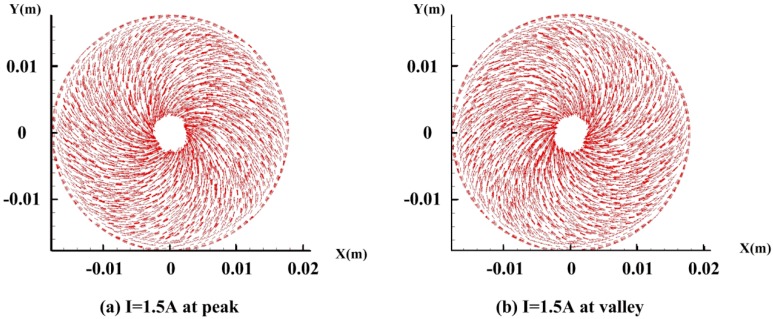
The vector graphs of velocity on the middle plane *z* = −0.001 m with the energized current *I* = 1.5 A at the peak and valley when rotating with Ω = sin(2π·0.01·t).

The vector graph of the velocity when the angular velocity reaches the peak and valley on the middle plane *z* = −0.001 m of the bottom fluid are presented in Figure 10 with energized current *I* = 1.5 A. The streamline is obviously controlled by the Coriolis effect irrespective of the direction of the rotation. The results provide compelling evidence that the Coriolis effect can be generated especially at low frequency in the C-MHD ARS, which is quite different from the laminar flow characteristics without energized current.

**Figure 11:**
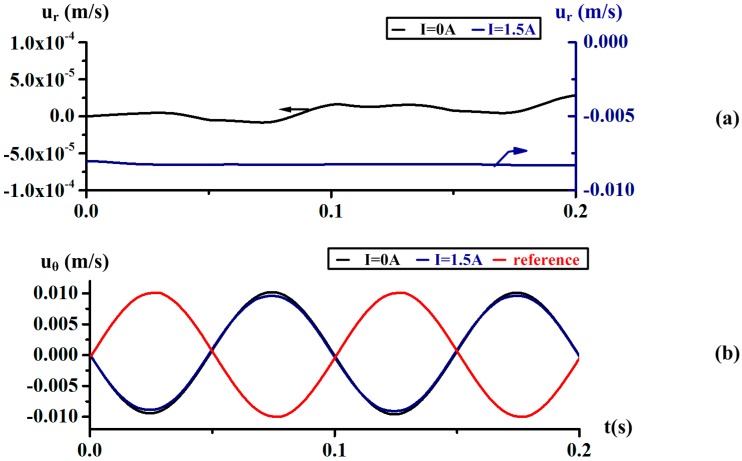
The radial velocity *u_r_* and relative circumferential velocity *u_θ_* of the test point *P*(0.010, 0, −0.001) in the sensing channel under an energized current *I* = 0 A and *I* = 1.5 A at the frequency *f* = 10 Hz.

In order to analyze the Coriolis effect varying with the rotating frequency, the simulation results at the frequency *f* = 10 Hz are shown in Figure 11. The radial velocity *u_r_* under energized current *I* = 0 is more negligible in contract to the results at low frequency in Figure 10, while the amplitude is also stable at 0.008 m/s under *I* = 1.5. The relative circumferential velocity *u_θ_* under I = 0 A and 1.5 A are almost and identical and both reverse to the reference signal. It seems that the fluid motion is rarely affected by the induced radial velocity. The phenomenon shows that the Coriolis effect is weakened when rotating at relatively high frequency in theory.

### 3.4. Simulation Results and Error Analysis of the Frequency Response of C-MHD ARS

**Figure 12:**
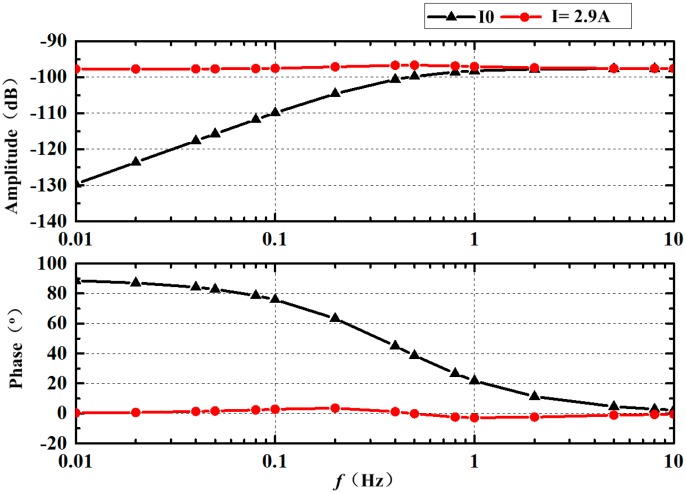
Frequency response simulation results of C-MHD ARS.

As mentioned earlier, it is difficult to set the exact value of the energized current for compensating according to the analytic expression. To investigate the appropriate frequency response through the whole bandwidth, we repeatedly tried to study the combined effect under the energized current. It is basic to give the frequency response of the sensing bottom channel without the energized current. The amplitude ratio and the phase differences between the voltage and angular velocity are calculated, when the reference rotating with Ω = sin(2π·*f*·t) at different frequencies. 500 intervals are set in every period for acquiring the phase accurately. Based on the modified simplified model analysis in our paper [12], the results plotted in Figure 12 are utilized to make fit the frequency domain to the method based on Levy’s work using the Gauss–Newton method for an iterative search. The pre-assigned orders of the numerator and denominator are respectively 1 and 1. The fitting results can be written as (18).

(18) $$H_{1}\left(s\right)=\frac{0.0000132s}{s+2.512}$$

Based on the modified analytic theory, the simplified expression (1) added modified factor *k* (0 < *k* < 1) can approximately be given. After substituting the geometric and physical parameters as listed in Table 1, the results can be written as (19) when the magnetic field *B* is set as the average value 0.12 T. As expected, the factor *k* is about 0.18 and the amplitude in simulation is smaller for the inhomogeneity of the magnetic field.

(19) $$\frac{\varphi (s)}{\Omega (s)}=\frac{0.000016s}{s+k\cdot 13.847+0.093}$$

Then energized current along the positive direction of the axis *y* is applied in the simulation. The current is increased gradually to improve the amplitude at *f* = 0.01 Hz until the ration is closed to 0.0000132. As presented in Figure 12, it is obvious that a significant improvement in the frequency response was obtained in the majority of cases. It should, however, be noted that the errors exist from 0.1 Hz to 2 Hz. When the energized current I is set at 2.9 A, the amplitude *f* = 0.01 Hz is a little smaller than the one at *f* = 10 Hz while the phase about 0°. As the rotating frequency accelerates, the amplitude increases until 0.2 Hz while the phase adds until 0.1 Hz. The amplitude peak at 0.2 Hz is 0.4 dB higher than that at 10 Hz, while the phase is about 3.5°. In the next, the amplitude and phase both decrease while the increase of frequency ranges from 0.2 Hz to 1 Hz, then the frequency response curve is close to a line above 1 Hz. Apparently, if the energized current continues to increase, the amplitude and phase at 0.01 Hz would be equal to the one at 10 Hz while the fluctuation in the transition zone would be larger. This indicates that the corner frequencies of the two effects of the Coriolis effect and MHD effect are a little different.

Thus, we tried to illustrate the compensated frequency response by the approximate analytic expression according to the fitting method based on Levy’s principle. The fitting result is written as (20) and its goodness of fit is up to 0.9. According to the record in simulation, the generated radial velocity under the energized current *I* = 2.9 A is about 0.015 m/s. Compared with the simplified model shown in Equation (2), we suppose that the corner frequency of the Coriolis effect in C-MHD ARS is higher than one of MHD effect.

(20) $$H_{2}\left(s\right)=\frac{0.0000132s}{s+2.512}+\frac{0.00001298*3.2}{s+3.2}$$

A specific analysis of the model errors will be given in the following section. In the moving reference frames of the rotating case, the relative circumferential velocity formulation of the Navier–Stokes equations takes the form of a non-inertial system along the circumferential direction:

(21) $$\frac{\partial u_{\theta }}{\partial t}+u_{r}\frac{\partial u_{\theta }}{\partial r}+u_{z}\frac{\partial u_{\theta }}{\partial z}+\frac{u_{r}u_{\theta }}{r}=f_{\theta }-\frac{\partial \Omega }{\partial t}-2\cdot \Omega \cdot u_{r}+\nu \left(\frac{\partial^{2}u_{\theta }}{\partial r^{2}}+\frac{1}{r}\frac{\partial u_{\theta }}{\partial r}-\frac{u_{\theta }}{r^{2}}\right)+v\frac{\partial^{2}u_{\theta }}{\partial z^{2}}$$

In simplifying the simple MHD ARS model, these terms $u_{r}\frac{\partial u_{\theta }}{\partial r},\;u_{z}\frac{\partial u_{\theta }}{\partial z},\;\frac{u_{r}u_{\theta }}{r},\;2\cdot \Omega \cdot u_{r}\;$ and $\nu \left(\frac{\partial^{2}u_{\theta }}{\partial r^{2}}+\frac{1}{r}\frac{\partial u_{\theta }}{\partial r}-\frac{u_{\theta }}{r^{2}}\right)$ may be approximately ignored when we suppose the velocity $\;u_{r},u_{\theta }$ are both far smaller than the circumferential velocity $u_{\theta }\;$ and the axis height *h* is negligible compared with the radial dimension *R*. As expected, the radial velocity $u_{r}$ is two orders of magnitude smaller than the circumferential velocity $u_{\theta }\;$ at relative low and high frequency with energized current *I* = 0, as seen in Figure 9 and Figure 11.

Nevertheless, the radial flow $u_{r}$ induced in C-MHD ARS is significant. As discussed above, the value radial velocity $u_{r}$ of 0.015 m/s evenly is comparable with the peak of the periodic circumferential velocity $u_{\theta }\;$. Therefore, the term $\frac{u_{r}u_{\theta }}{r}$ should be taken into consideration when the Coriolis effect plays a leading role at low frequency. The simplified model (2) could be re-written as (22), which is consistent with the fitting results obtained in simulation shown in Equation (20) above. It is believed, in theory, that the fluctuation exists in the transition zone in combing the Coriolis and MHD effect.

(22) $$\left|\frac{\varphi \left(s\right)}{\Omega \left(s\right)}\right|=\frac{B_{z}WRs}{s+\nu /h^{2}+k\cdot \sigma {B_{z}}^{2}/\rho }+\frac{B_{z}W\cdot 2u_{r}}{s+\nu /h^{2}+k\cdot \sigma {B_{z}}^{2}/\rho +u_{r}/R}$$

## 4. Experimental Results

**Figure 13:**
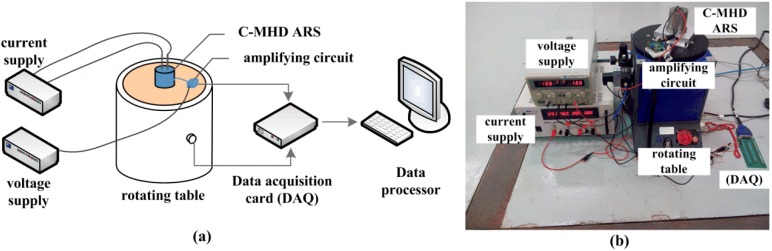
Frequency response experiment designed for C-MHD ARS. (**a**) Sketch Map (**b**) Physical map.

As outlined in the Section 3.1, the C-MHD ARS is designed and assembled to make an experiment that is comparable with the simulation results. In this work, we sought to provide the frequency response of a MHD ARS combing with the Coriolis effect at low frequency. The sensor is an open-loop system and works in a wide band, so the sweep-frequency test on a rotating table was adopted as the simple MHD ARS. In particular, a current supply connected to the energized electrodes is needed to drive the MHD pump in the C-MHD ARS. A large current should be provided by the current supply, although the power requirement is relatively low for extremely small impedance of the fluid in MHD pump. As discussed above, the current set for compensating could not be acquired in advance and needs to be tested again and again according to the experimental results at low frequency. Besides, the amplifying circuit is redesigned to improve the low-frequency noise suppression for the bandwidth extension at low frequency and still needs a voltage source to supply power. The magnification is set to 10,000. The data acquisition card (DAQ) and data process are adapted to collect the signal of the C-MHD ARS and the applied vibration by the rotating table. The sketch and physical map of the sweep-frequency experiment are presented in Figure 13.

**Figure 14:**
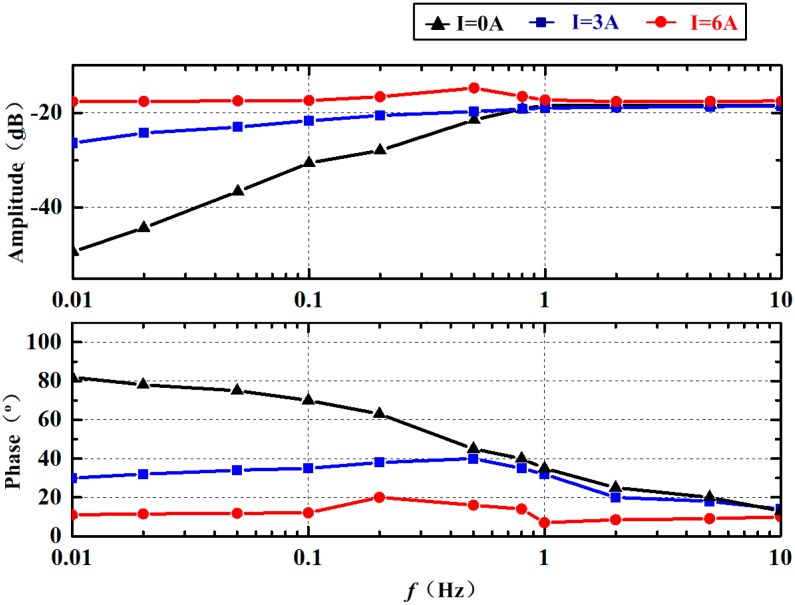
Frequency response experimental results of C-MHD ARS.

In the beginning, the frequency response of the sensor without the energized current is established and the results are plotted in Figure 14. The fixed phase deviation of about 10° is caused by the phase difference of the amplifier circuit. The corner frequency in the experiment is about 0.5 Hz, which is consistent with the one in the simulation presented in Figure 12. Besides, the amplitude after amplified is also in good agreement with the simulation results. Our results provide compelling evidence that the simulation method and the modified analytic model can be applied in the MHD ARS study. Then, the energized current is set to 3 A according to the numerical simulation. The experimental results of the frequency response at *I* = 3 A are illustrated in Figure 14. It seems that the frequency response at low frequency has been improved but is far from the ideal. Unfortunately, the increase of amplitude and the reduction of phase difference are both relatively insufficient. The deviation of the frequency response in the experiment from the simulation results may be attributed to the weakened magnetic field intensity in the MHD pump and the viscous force and friction of the fluid channel in practice.

In the next experiment, the energized current is increased slowly until the value is set to 6 A. As displayed in Figure 14, the amplitude and phase at *f* = 0.01 Hz are a little smaller than the one at *f* = 0.01 Hz. A similar trend can be observed in the range from 0.01 Hz to 10 Hz compared with the simulation results in Figure 12. The amplitude increases from 0.01 Hz to 0.5 Hz and then decreases to the value without an energized current until 2 Hz. As displayed, the amplitude at about *f* = 0.5 Hz reaches the peak, which is larger than the ratio above 1 Hz. Meanwhile, the phase fluctuation appears to mainly range from 0.1 Hz to 10 Hz. As shown, the phase gradually rises to deviate from the ideal value, then gradually falls below the ideal value and finally returns to the ideal value. The phenomena strongly confirmed that the error in the combination with the Coriolis and MHD effect exists, as illustrated in Section 3.4. It is difficult to acquire the ideal frequency characteristic curve directly by applying a fixed energized current in the MHD pump. The compensation method needs to be further studied. Furthermore, the energized current needed in the C-MHD ARS in this paper is less than perfect for the high density and dynamic viscosity of Galinstan. The design should be updated until the energized current is allowable.

## 5. Conclusions

In this paper, the model of the MHD ARS combined with the Coriolis effect is studied, which is pivotal to extend the sensor’s measurement scope throughout the whole bandwidth. Firstly, a comprehensive solving method of interaction between the flow field and the electromagnetic field suitable for the C-MHD ARS is proposed based on the conventional magnetic and electric approach. Then, the C-MHD ARS with an MHD pump in the middle fluid channel is designed. A numerical simulation of the MHD pump, Coriolis effect, and frequency response in the C-MHD ARS is made. Finally, the corresponding error analysis and experiment results are given to confirm the simulation results. The main conclusions are as follows:

The uniform radial velocity in the sensing channel could be induced when the flow is driven upward in the MHD pump. Then the Coriolis effect can be generated and is weakened when the rotating frequency increases.

Notably, the performance of the MHD ARS at low frequency can be improved by introducing the Coriolis effect at low frequency. However, the corner frequencies of the two effects of the Coriolis effect and MHD effect are a little different, which may cause the fluctuation in the transition zone of the frequency response.

The error source of the two effects combination may mainly be attributable to the item *u_r_*/*R* in the modified simplified C-MHD, which should be minimized in the sensor design. It is difficult to acquire the ideal frequency characteristic curve directly by applying a fixed energized current in the MHD pump.

Our study provides an error analysis method of MHD ARS combined with the Coriolis effect and offers a framework for future studies to minimize the error. In the future, the compensation signal processing method will be studied and a suitable closed-loop control method of the energized current will be researched. Besides, the reliability of the angular position and the acceleration measured by the MHD ARS should be studied deeply to expand its application in vibration testing and navigation.

## Figures and Tables

**Table 1:** Main mechanical structure parameters and physical parameters of the fluid channel.

Mechanical Structure Parameters	(mm)	Physical Parameters of Conducting Fluid	Galinstan
Inner radius of annular channel	4	density *ρ* (kg/m^3^)	6.4 × 10^3^
Outer radius of annular channel	14.5	dynamic viscosity *υ*·*ρ* (kg/cm^3^)	0.0024
Height of upper and bottom channel	2	electric conductivity *σ* (s/m)	3.46 × 10^6^
Thickness of outside channel	2	Magnetic permeability *μ* (H/m)	1.257 × 10^−6^
Thickness of central channel	2	melting point (°C)	−19
Width of central channel	10	boiling point (°C)	>1300
Total height	37	Gravity	√
